# Wild bees preferentially visit *Rudbeckia* flower heads with exaggerated ultraviolet absorbing floral guides

**DOI:** 10.1242/bio.20146445

**Published:** 2014-02-28

**Authors:** Lisa Horth, Laura Campbell, Rebecca Bray

**Affiliations:** Department of Biological Science, 4700 Elkhorn Avenue, Old Dominion University, Norfolk, VA 23529, USA

**Keywords:** Bee, *Rudbeckia*, Flower, Pollination, Opsin, Vision, Pollinator

## Abstract

Here, we report on the results of an experimental study that assessed the visitation frequency of wild bees to conspecific flowers with different sized floral guides. UV absorbent floral guides are ubiquitous in Angiosperms, yet surprisingly little is known about conspecific variation in these guides and very few studies have evaluated pollinator response to UV guide manipulation. This is true despite our rich understanding about learning and color preferences in bees. Historical dogma indicates that flower color serves as an important long-range visual signal allowing pollinators to detect the flowers, while floral guides function as close-range signals that direct pollinators to a reward. We initiated the work presented here by first assessing the population level variation in UV absorbent floral guides for conspecific flowers. We assessed two species, *Rudbeckia hirta* and *R. fulgida*. We then used several petal cut-and-paste experiments to test whether UV floral guides can also function to attract visitors. We manipulated floral guide size and evaluated visitation frequency. In all experiments, pollinator visitation rates were clearly associated with floral guide size. Diminished floral guides recruited relatively few insect visitors. Exaggerated floral guides recruited more visitors than smaller or average sized guides. Thus, UV floral guides play an important role in pollinator recruitment and in determining the relative attractiveness of conspecific flower heads. Consideration of floral guides is therefore important when evaluating the overall conspicuousness of flower heads relative to background coloration. This work raises the issue of whether floral guides serve as honest indicators of reward, since guide size varies in nature for conspecific flowers at the same developmental stage and since preferences for larger guides were found. To our knowledge, these are the first cut-and-paste experiments conducted to examine whether UV absorbent floral guides affect visitation rates and pollinator preference.

## INTRODUCTION

A comprehensive, high quality literature exists regarding the sensory and cognitive abilities of bees. This research includes extensive data on essential tasks associated with foraging, like visual choice, learning and memory (e.g. von Frisch, 1914; Daumer, 1956; Daumer, 1958; Backhaus, 1991; Giurfa et al., 1996; Dyer et al., 2008; Dyer et al., 2011; Spaethe et al., 2001). Bees and humans both have trichromatic vision, but bee vision is short wave shifted compared to humans. Retinal photoreceptors of bees are short (SWS), medium (MWS), and long wavelength sensitive (LWS) and are also classified as ultraviolet (UV), blue, and green sensitive. Data from 43 species of hymenoptera demonstrate maximal receptor sensitivity (λ max) at ∼340 nm, 430 nm, and 535 nm, respectively (Peitsch et al., 1992; Menzel and Blakers, 1976; Menzel and Backhaus, 1991 and references therein).

The color space perceived by bees has been determined using mathematical modeling and multidimensional scaling analysis that employed empirical color-choice test results (Backhaus et al., 1987; Backhaus, 1991; Chittka et al., 1992). The color differences potentially detectible by bees were calculated for different chromatic dimensions (e.g. uv/blue-green and blue/uv-green axes) (Backhaus, 1991). Neurons with the same photoreceptor antagonism have been identified in the medulla and lobula of the bee brain (Kien and Menzel, 1977a; Kien and Menzel, 1977b). The spectral sensitivities predicted for hypothetical color opponent coding (COC) cells compared well to empirical measures for antagonistically coding neurons (Backhaus, 1991).

For bees to be able to detect flowers in the distance, contrast between floral spectral reflectance and background reflectance is necessary (e.g. Kevan, 1978; Chittka and Menzel, 1992). Green (LWS) receptor contrast is particularly important for distance-based detection, which is believed to occur prior to chromatic contrast (Giurfa et al., 1996). Green contrast is important for motion processing (Srinivasan and Lehrer, 1984) and detection of object edges (Lehrer et al., 1990). In honeybees, green contrast is detected by LWS receptors when the visual angle is small (5–15°), whereas chromatic traits are detected by SWS and MWS receptors at greater visual angles (>15°) (Giurfa et al., 1997; Giurfa and Vorobyev, 1998; Spaethe et al., 2001; Dyer et al., 2011).

From an evolutionary perspective, a trade off was found between foraging accuracy and decision time for bumble bees (*Bombus* L. *terrestris*). When foraging in a virtual flower meadow where ‘flowers’ had similar colors but different rewards, bees were more accurate when they took longer to make foraging choices (Chittka et al., 2003). Fast and slow bees did learn to slow down and improve accuracy with aversion stimuli (Chittka et al., 2003). However, fast bees had higher nectar collection rates (Burns, 2005), so costs are associated with slow, accurate decision-making.

In nature, competition for pollination is known to contribute to natural selection on floral traits (Caruso, 2000) and visual cues clearly affect conspecific floral attractiveness to pollinators. Field studies have shown that yellow flowered wild radishes (*Raphanus raphanistrum* L.) had higher pollination rates than white ones (Stanton et al., 1989) and deep blue flowered montane larkspur (*Delphinium nelsonii* Greene, *Delphinium nuttallianum* Pritz. ex Walp) had higher pollination rates than albinos (Waser and Price, 1981). Recent work with model flowers showed that bumble bees (*B. terrestris*) have innate preference for high spectral purity (Rohde et al., 2013). The value of colorful floral traits that we see is less contentious than the value of traits invisible to us but visible to bees.

The scientific community has ranged the gamut from intense interest in UV traits (Bennett and Cuthill, 1994), to discounting what was considered exaggerated attention and promoting the idea that UV is not a special channel for communication (e.g. Chittka et al., 1994). Middle ground was reached with demonstrations that floral UV reflection can be important, though sometimes no more important than blue, green or red reflection (Kevan et al., 2001). Despite the fact that UV reflective and absorptive floral patterns are widespread in nature and visible to pollinators (von Frisch, 1967; Daumer, 1958; Eisner et al., 1969; Silberglied, 1979; Höglund et al., 1973; Burkhardt, 1982; McCrea and Levy, 1983; Peitsch et al., 1989; Menzel and Backhaus, 1991; Chittka and Menzel, 1992; Peitsch et al., 1992; Bennett et al., 1996; Dyer, 1996; Briscoe et al., 2003; Winter et al., 2003; Skorupski and Chittka, 2010) few studies have empirically manipulated UV floral guides to assess bee visitation frequency or preference.

Early on Daumer elucidated the point that primary colors combine to create ‘novel’ colors visible to bees but not humans (Daumer, 1956; Daumer, 1958; see also Backhaus, 1991; Giurfa et al., 1995). Later, when Waser and Price were studying foraging economics, they painted albino montane larkspurs (Waser and Price, 1985). They showed that adding UV absorbent blue paint to albino sepals and guard petals that typically reflect UV enhanced visitation and decreased foraging time. So did performing this manipulation on albino nectariferous petals that typically absorb UV, making this a blue-only manipulation (Waser and Price, 1985).

Burr et al. later discussed the function of UV reflection as a long-range landing site cue (Burr et al., 1995). Then Eisner illustrated how ‘human-yellow’ on the distal half of *Rudbeckia hirta* petals was UV reflective (Eisner, 2002) and referred to this as ‘bee purple’ (originally described by Daumer, 1956; Daumer, 1958). Around this time Johnson and Anderson conducted field studies with the African potato (or star grass, *Hypoxis hemerocallidea* Fisch. & C. A. Mey) and showed that, when floral UV reflectance was obscured with sunscreen, fewer honey bees (*A. mellifera scutella*) approached and landed on flowers (Johnson and Andersson, 2002).

Burr et al. also discussed the use of very small UV absorptive areas on flowers as guides for orientation to reward (Burr et al., 1995). Such orientation cues have been called honey- or nectar-guides and will hereafter be referred to as floral guides. UV absorbance is quite common in these floral guides. In a tropical field study, UV absorbent banner petals were repositioned on legume (*Caesalpinia eriostachys* Benth. and *Parkinsonia aculeata* L.) flowers to successfully demonstrate their orientation function (Jones and Buchmann, 1974). Chemically, flavonol glucosides are primarily responsible for the large, UV absorbing floral guide in the floral heads of some asters, including *Rudbeckia hirta* L. (Thompson et al., 1972; Schlangen et al., 2009), one of the species used in the work we present here.

Our studies involved wild, cultivated and empirically manipulated asters (*R. hirta* and *R. fulgida*). First, we report novel findings regarding the distribution of floral guide sizes in natural populations and cultivars. We hypothesize that UV absorbent floral guides are important for attracting pollinators, not just for orientation, and that larger guides will be more attractive than smaller ones. To test these ideas we compare pollinator visitation rates after enlarging and diminishing natural UV-absorbent orientation cues. We focus on Halictidae (sweat bees), the relatively poorly studied, yet numerically abundant, cosmopolitan generalist pollinators frequently found in urban ecosystems (Dikmen, 2007). This work was completed using a ‘cut-and-paste’ design akin to Andersson's widowbird tail-length studies (Andersson, 1982). This is a timely demonstration that the size of UV absorbent floral guides clearly affects native bee visitation rates and that these floral guides may not be used solely for orientation toward a reward, as once believed, but also play a role in pollinator recruitment.

## RESULTS

### I. Floral specimens and relevant techniques for measuring UV floral guides

**Fig. 1.**
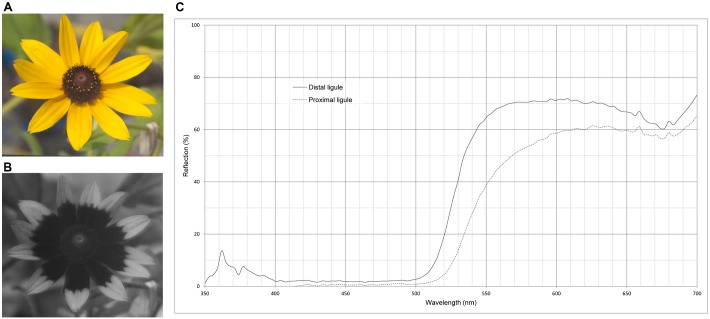
Spectrophotometric reflectance. (A) Native *Rudbeckia hirta* as seen with color photography. (B) Native *R. hirta* as seen with ultraviolet (UV) photography. (C) Spectrophotometric reflectance plot of the proximal (…) and distal (—) portions of the *R. hirta* ligules, or petals. Relative reflection (%) is plotted as a function of wavelength (nm).

Color and ultraviolet absorbing *Rudbeckia hirta* photographs are presented in Fig. 1A,B, along with the spectrophotometric graph of floral reflectance (Fig. 1C) showing the different reflectance patterns in the distal and proximal portions of *R. hirta* ligules. Relevant measurements and the photographs of ‘cut-and-paste’ treatments for each experiment are presented in the associated Results sections below.

### II. Floral guide size-distributions for three data sets: greenhouse, urban, and wild *R. hirta*

#### Greenhouse flowers

The average UV floral guides in greenhouse plants covered 62% (standard error 1.3%) of the petal length for flowers from the first plant and 57% (standard error 0.8%) of the petal length for flowers from the second plant. These averages differed (T = 3.24, d.f. = 16, and P = 0.005) despite the plants' shared environmental conditions. Floral guide size variation was greater between plants than for flowers on a single plant.

#### Naturalized urban flowers

The average UV floral guide in the naturalized plants covered 51% (standard error 1.23%) of the total petal surface area. However, there was a 27% range in this estimate, with a min–max of 39–66%. A strong correlation existed between floral head size and floral guide size (r = 0.928, *n* = 25, P<0.001). This simply means that bigger flowers had more UV absorptive petal surface area than littler flowers, which says nothing about the relative size of floral guides. Perhaps more compelling is the result that arises when we address the relative amount of the petal surface that is comprised of floral guide. No correlation existed between the flower head size and the proportion of UV absorptive petal surface area (r = 0.051, *n* = 25, P = 0.810). In other words, larger flowers did not have relatively larger floral guides than smaller flowers.

#### Wild flowers

The average UV floral-guide in the wild plants covered 44% (standard error 1.69) of the total petal surface area. This was 7% smaller than the urban population average guide size. However, there was a 39% range in this estimate, with a min–max of 26–64%. This range was 11% more than the urban population range. Like the urban population, no correlation existed between the floral head size and the proportion of UV absorptive petal surface area (r = 0.191, *n* = 21, P = 0.431). Meaning once again, larger flowers did not have relatively larger floral guides than smaller flowers.

### III. Insect visitation response to floral guide manipulations

#### a. Sunscreen masked floral guides on *R. hirta*

**Fig. 2.**
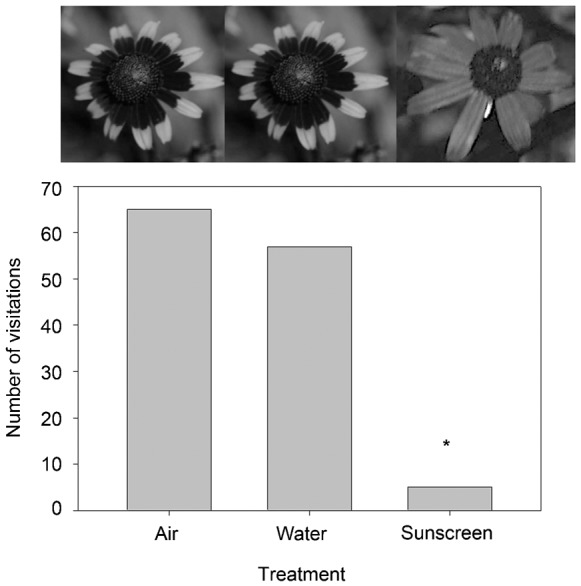
Results of a sunscreen experiment on the grounds of an urban campus. The *R. hirta* floral head treatment UV photographs are displayed atop each data bar. Note the masking of the lack of bold UV absorbent floral guide in the sunscreen treatment. The asterisk represents a significant difference in the number of visitors to the ‘sunscreen’ treatment relative to the other treatments.

Sunscreen obscured the floral guide typically found on *R. hirta* petals, and diminished UV reflection too (Fig. 2). The fine mist sunscreen dried quickly and left treated flowers dry. Fewer insects visited the sunscreen treatment than the other treatments. A total of 127 insects were observed visiting flowers in this experiment (five visited the ‘sunscreen’ treatment, 57 visited the ‘water misted’ treatment and 65 visited the ‘air sprayed’ treatment; Table 1). The Chi-square test was highly significant refuting the null hypothesis of an equal distribution of visitors among treatments (χ^2^_(0.05,2)_ = 50.1, P<0.001, two tail test). Post-hoc pairwise comparison tests indicated a difference between the number of visitors to the ‘sunscreen’ treatment when compared to the ‘water misted’ (Δχ^2^ = 27.85>3.8, P<0.05), and the ‘air sprayed’ treatment (Δχ^2^ = 20.80>3.8, P<0.05). The community of visitors was diverse and included the following 10 families: Scoliidae (*n* = 45), Apidae (28), Halictidae (18), Syrphidae (13), Megachilidae (13), Hesperiidae (2), Sphecidae (3), Culicidae (1), Muscidae (2), Vespidae (1) and an unidentified fly (1). Since Scoliidae are generally considered minor pollinators, removing this family, as well as the single visitors that were not likely major pollinators (e.g. Culicidae), and repeating the test did not affect the outcome. Visitors to the ‘sunscreen’ treatment included four families: Syrphidae (1), Megachilidae (1), Muscidae (2) and Vespidae (1).

**Table 1.**
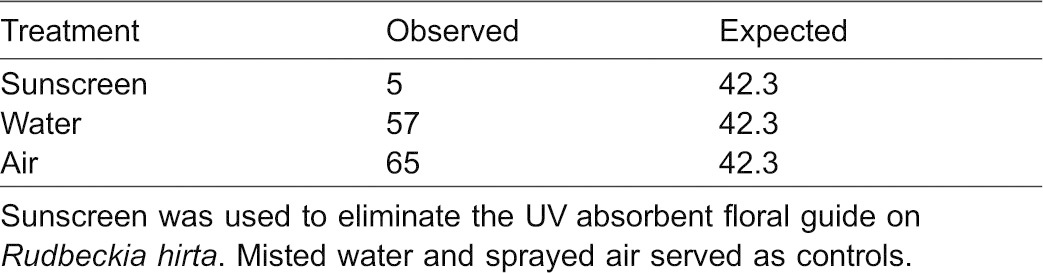
Results from Experiment IIIa

#### b. Augmented floral guides on *R. fulgida*

**Fig. 3.**
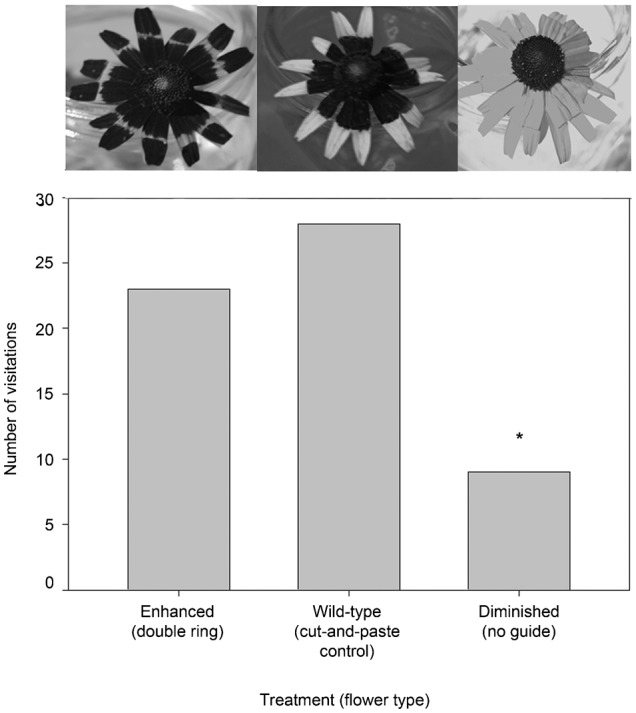
Results of an urban home garden experiment. The *R. fulgida* floral head treatment UV photographs are displayed atop each data bar. Here, the ‘enhanced’ floral guide creates two concentric rings of UV absorbance, and the ‘diminished (no guide)’ treatment lacks the classic UV absorbing floral guide. The asterisks represent a significant difference in the number of visitors to the ‘enhanced’ treatment relative to the ‘diminished’ treatment.

A total of 60 insects were observed visiting flowers in this experiment (23 visited the ‘enhanced’ (double ring) floral guide treatment, nine visited the ‘diminished’ (no guide) treatment, and 28 visited the ‘wild-type cut-and-paste control’; Table 2; Fig. 3). The Chi-square test was highly significant refuting the null hypothesis of an equal distribution of visitors among treatments (χ^2^_(0.05,2)_ = 9.7, P = 0.008, two tail test). This result was not attributable to a positional effect of the treatments (i.e. flower location in array, χ^2^_(0.05,2)_ = 0.7, P = 0.705). Post-hoc pairwise comparison tests indicated a difference between the number of visitors to the ‘enhanced’ treatment when compared to the ‘diminished’ treatment (Δχ^2^ = 5.6>3.8, P<0.05). Post-hoc results might appear counterintuitive, because the test is based on a squared deviation from the expectation. The squared deviation was smallest for the ‘enhanced’ treatment since it deviated least from the expected value, making the Δχ^2^ largest when comparing this treatment to the ‘diminished’ treatment because it had the greatest deviation from the expected value. The frequency of visitors to the ‘enhanced’ treatment increased over time but decreased for the control (after Day 1). Frequency of visitors for Days 1 to 6 to the ‘enhanced’ treatment were: 0.250, 0.140, 0.160, 0.375, 0.500, 0.625, and to the ‘control’: 0.633, 0.714, 0.600, 0.375, 0.375, 0.250).

**Table 2.**
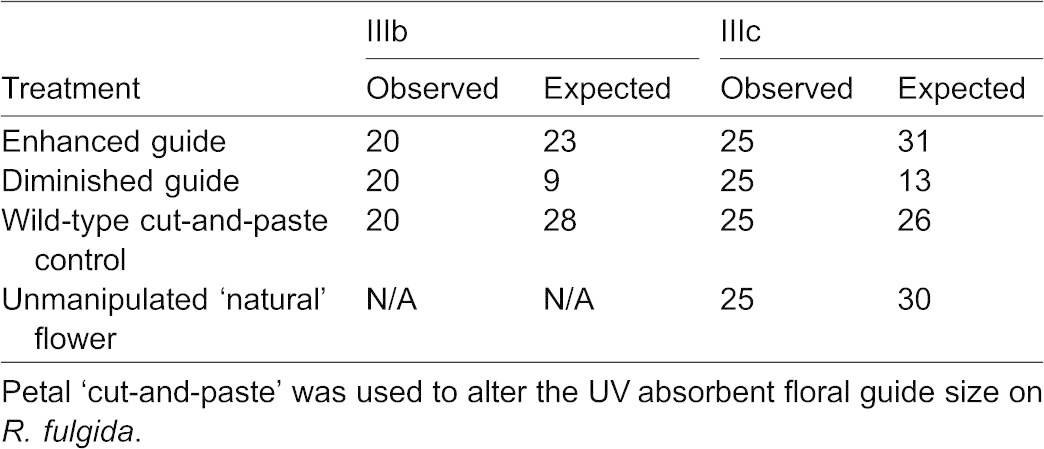
Results of Experiments IIIb,c

The community of visitors to this home garden experiment was less diverse than to the urban campus experiment. Here, Halictidae (sweat bees) comprised 75% (45/60) of the visitors and the number of their visits was similarly distributed across treatments each day. Substantially fewer individuals visited this garden from seven additional families: Papilionidae (3), Scoliidae (3), Apidae (2), Syrphidae (2), Megachilidae (2), Sphecidae (2), and Hesperiidae (1).

#### c. Slightly enlarged and diminished floral guides on *R. fulgida*

**Fig. 4.**
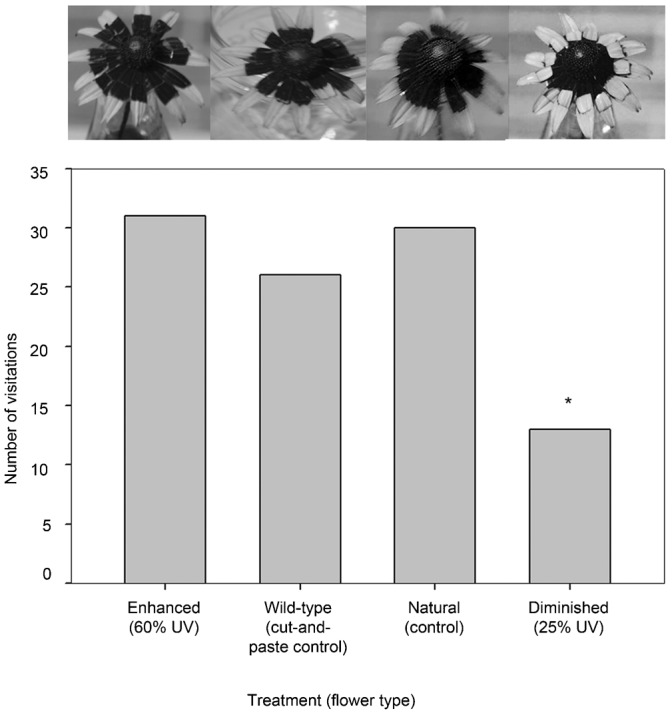
Results of an urban preserve experiment. The *R. fulgida* floral head treatment UV photographs are displayed atop each data bar. Here the ‘enhanced’ floral guide treatment is slightly enlarged, with ∼60% of the petal surface area UV absorbent and the ‘diminished’ floral guide treatment has ∼25% of the petal surface area UV absorbent. The asterisk represents a significant difference in the number of visitors to the ‘diminished (25% UV)’ treatment relative to the other treatments.

A total of 100 insects were observed visiting flowers in this experiment (31 visited the ‘enhanced’ (UV absorbent guide was ∼60% of petal surface area) floral guide treatment, 13 visited the ‘diminished’ (∼25%) floral guide, 26 visited the ‘wild-type cut-and-paste control’, and 30 visited the ‘natural’ flower; Table 2; Fig. 4). All insects were either major or minor pollinators and therefore included in the analysis. The Chi-square test was significant refuting the null hypothesis of an equal distribution of visitors among treatments (χ^2^_(0.05,3)_ = 8.2, d.f. = 3, P = 0.041). Post-hoc pairwise comparison tests indicated a difference in the number of visitors to the ‘diminished’ treatment and all other treatments (Δχ^2^ = 4.76>3.84, P<0.05; Δχ^2^ = 5.74>3.84, P<0.05; Δχ^2^ = 4.32>3.84, P<0.05). The community of visitors was diverse and included the following nine families: Apidae (33), Halictidae (33), Hesperiidae (9), Lycaenidae (6), Nymphalidae (6), Syrphidae (6), Pieridae (2), Hesperiidae (2), and Scoliidae (1).

#### d. Oversized floral guides on *R. hirta*

**Fig. 5.**
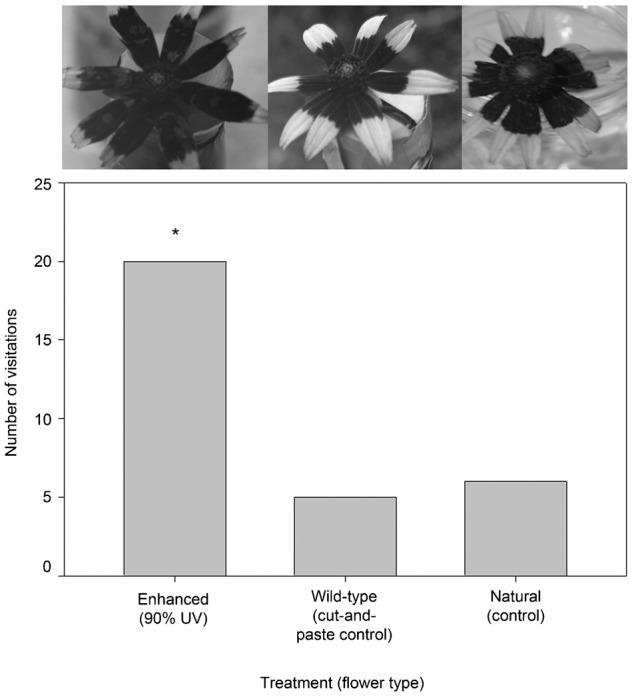
Results of an urban zoo grounds experiment. The *R. hirta* floral head treatment UV photographs are displayed atop each data bar. Here the ‘enhanced’ floral guide treatment is very large, with ∼90% of the petal surface area UV absorbent. The asterisk represents the significant difference in the number of visitors to the ‘enhanced (90%)’ treatment relative to the other treatments.

In June, a total of 31 insects were observed visiting flowers in this experiment (20 visited the ‘enhanced’ (90%) floral guide, five visited the ‘wild-type cut-and-paste control’, and six visited the ‘natural’ flower; Table 3; Fig. 5). The Chi-square test was highly significant refuting the null hypothesis of an equal distribution of visitors among treatments (χ^2^ = 13.6, P = 0.0011). Post-hoc pairwise comparison tests indicated a difference in the number of visitors to the ‘enhanced’ treatment compared to the ‘wild-type cut-and-paste control’ and ‘natural’ flower (Δχ^2^ = 6.30>3.84, P<0.05 and 7.24>3.84, P<0.05). The total visitation times were 472 seconds to the ‘enhanced’ treatment, 50 seconds to the ‘wild-type cut-and-paste control’ and 62 seconds to the ‘natural’ flower. Mean visitation times were 23.5 (s.d. = 27.0), 10.0 (s.d. = 9.59) and 10.3 (s.d. = 9.11) seconds, respectively. ANOVA analysis indicated that mean visitation times were not significantly different (Sums of Squares (SS) between groups = 8.8088, SS within groups = 70.54, Total = 78.6, d.f. = 2, 27 and 29, respectively; Mean Square (MS) between groups = 4.04, within group 2.61, F = 1.548 and P = 0.231). The community of visitors decreased in diversity and included two families: Halictidae (30), and Apidae (1).

**Table 3.**
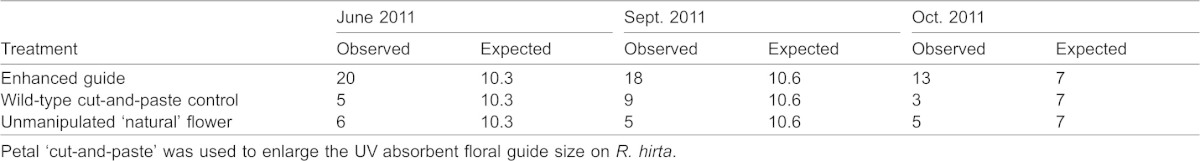
Results of Experiment IIId

**Fig. 6.**
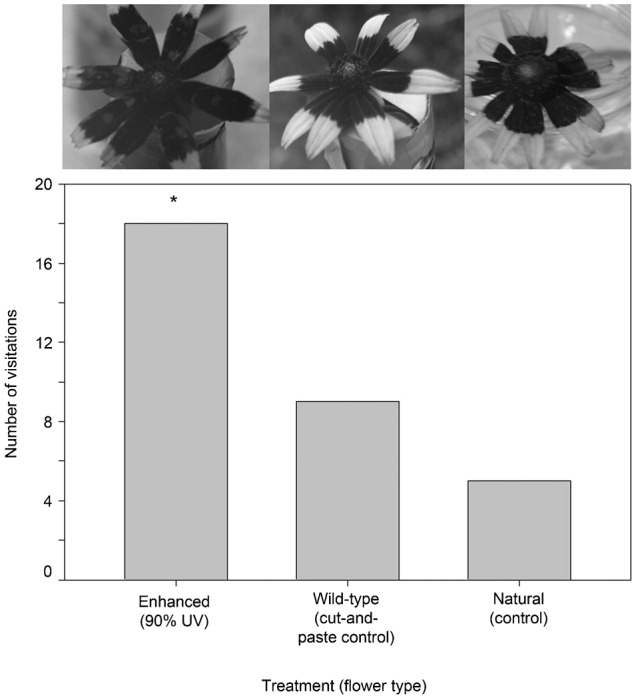
Results of an urban zoo grounds experiment. The *R. hirta* floral head treatment UV photographs are displayed atop each data bar. Here the ‘enhanced’ floral guide treatment is very large, with ∼90% of the petal surface area UV absorbent. The asterisk represents the significant difference in the number of visitors to the ‘enhanced (90%)’ treatment relative to the ‘control’ flower.

In September, a total of 32 insects were observed visiting flowers in this experiment (18 visited the ‘enhanced’ floral guide, nine visited the ‘wild-type cut-and-paste control’ and five visited the ‘natural’ flower; Table 3; Fig. 6). The Chi-square test was again highly significant refuting the null hypothesis of an equal distribution of visitors among treatments (χ^2^ = 8.3, P = 0.016). Post-hoc pairwise comparisons again indicated a difference in the number of visitors to the ‘enhanced’ treatment compared to the ‘wild-type cut-and-paste control’ (Δχ^2^ = 4.78>3.84, P<0.05), but not to the ‘natural’ flower (Δχ^2^ = 2.03<3.84, P>0.05). The total visitation times were 192 seconds to the ‘enhanced’ treatment, 75 seconds to the ‘wild-type cut-and-paste control’ and 71 seconds to the ‘natural’ flower. Mean visitation times were 10.6 (s.d. = 13.6), 8.22 (s.d. = 7.34), and 14.6 (s.d. = 20.0) seconds, respectively. ANOVA analysis indicated that mean visitation times were not significantly different (SS between groups = 0.081, SS within groups = 4.93, Total = 5.01, d.f. = 2, 29 and 31, respectively. MS between groups = 0.040, within group 0.170, F = 0.237 and P = 0.791). The community of visitors included three families: Halictidae (17), Apidae (12) and Syrphidae (3).

**Fig. 7.**
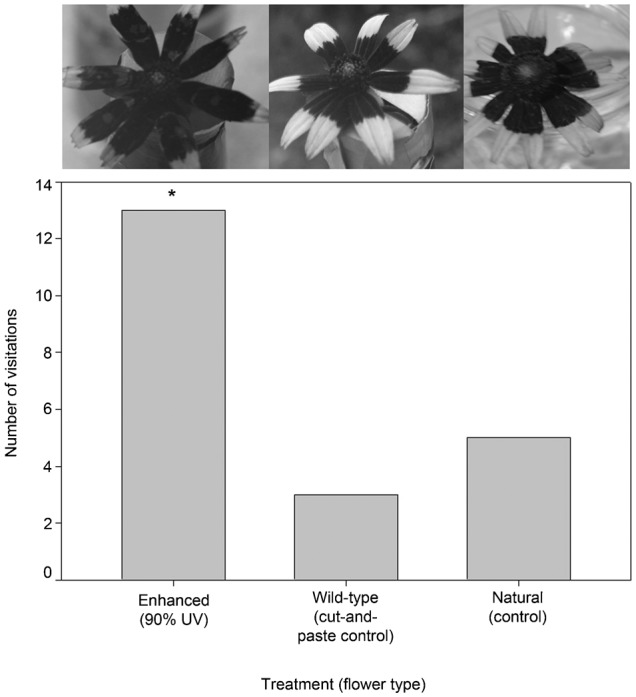
Results of an urban zoo grounds experiment. The *R. hirta* floral head treatment UV photographs are displayed atop each data bar. Here the ‘enhanced’ floral guide treatment is very large, with ∼90% of the petal surface area UV absorbent. The asterisk represents the significant difference in the number of visitors to the ‘enhanced (90%)’ treatment relative to the ‘natural’ flower.

In October, a total of 21 visitors were observed visiting flowers in this experiment (13 visited the ‘enhanced’ floral guide, three visited the ‘wild-type cut-and-paste control’ and five visited the ‘natural’ flower; Table 3; Fig. 7). The Chi-square test was again highly significant refuting the null hypothesis of an equal distribution of visitors among treatments (χ^2^ = 8.0, P = 0.018). Post-hoc pairwise comparison tests indicated a difference in the number of visitors to the ‘enhanced’ treatment compared to the ‘natural’ flower (Δχ^2^ = 4.57>3.84, P<0.05) but not to the ‘wild-type cut-and-paste control’ (Δχ^2^ = 2.86<3.84, P<0.05). The community of visitors increased slightly in diversity compared to the two prior experiments due to an influx of butterflies and included five families: Halictidae (15), Nymphalidae (2), Pieridae (2) Papilionidae (1), and Apidae (1). The one-day food color mark recapture study yielded no repeat visitors on Day 2 but captive insects retained their color.

## DISCUSSION

Despite the ubiquity of UV absorbent floral guides in Angiosperms, very little work has addressed the association between pollinator visitation frequency and UV floral guide traits, possibly because these traits were assumed to serve orientation purposes only. We show clearly that wild bees are more frequently attracted to *Rudbeckia* with oversized UV absorbing floral guides than to conspecific flowers with guide sizes closer to the average found on wild and cultivated flowers. Bees also demonstrate less recruitment to flowers with very small guides. We also found that sunscreen treated flowers with masked UV traits were avoided by pollinators, which is consistent with prior work of the same nature (Johnson and Andersson, 2002). However, we did not control for potential alternative chemosensory cues (e.g. sunscreen scent) so the sunscreen experiment must be interpreted with caution.

Our work shows that floral guides functioned in distance-based recruitment. Preference for relatively large floral guides held true across our experiments despite the fact that in nature, bigger flowers do not have relatively larger floral guides than smaller flowers. Daumer (Daumer, 1956; Daumer, 1958) and others (e.g. Burr et al., 1995) posited that UV reflectance attracts pollinators from a distance and that floral guides are used for close-range reward-orientation. While this is true, and could perhaps be particularly pertinent to floral species without radial symmetry, or with particular types of floral guides, we have also shown that in *Rudbeckia*, when we decrease the proportion of the floral head surface area that is UV reflective and increase the size of the UV absorbent guides, we see higher recruitment.

Chittka et al. have shown that pure UV reflecting flowers are very rare (Chittka et al., 1994). They comprised less than 4% of over 500 species surveyed. It is logical, based upon what we know about insect vision, that purely UV reflecting flowers would not be attractive to bees. Chittka et al. found that if flowers reflected UV they also tended to reflect red (Chittka et al., 1994), which may aid in recruitment of some insect pollinators. The reflectance curves for ∼17% of their surveyed flowers, resemble our data for the proximal half of the *Rudbeckia* petal surface (e.g. human yellow and UV absorptive). Chittka et al. label this reflectance pattern ‘green’ for bees (Chittka et al., 1994). The reflectance curves for ∼13% of their surveyed flowers resemble our data for the distal half of the *Rudbeckia* petal surface (e.g. human yellow and UV reflective). Chittka et al. label this pattern ‘UV-green’ for bees (Chittka et al., 1994). These reflectance spectra are predictably distinguishable from environmental background material including leaves, soil and rock, much of which may appear more gray to bees (Daumer, 1958; Kevan, 1978; Menzel and Shmida, 1993).

The visitation preferences that we identified are generalizable for at least three floral species (*R. hirta*, *R. fulgida*, and unpublished data for *Ranunculus bulbosus* L. Ranunculaceae). Bees are capable of learning fine color differences through trial and error (Reser et al., 2012); however, the minimal variation in ‘human yellow’ seen between the distal and proximal portions of the *Rudbeckia* petals are not predicted to have driven our results for untrained bees where we did not link this difference to reward or aversion resources. Bees generally take longer to discriminate between similar colors than dissimilar ones and the neural pathway invoked for learning fine differences demonstrates plasticity, unlike the pathway for coarse color discrimination, which functions more rapidly and is hard-wired (Dyer et al., 2011). The wild bees recruited in our work may have had prior experience in nature. So, we purposefully used multiple urban settings with unique surrounding vegetation, including different floral species at different experimental sites, so that prior learning would not bias our results. However, additional research, perhaps using ‘cut-and-paste’ style treatments with UV absorbent petals from another species, placed atop *Rudbeckia* flowers, might reinforce our results and eliminate the confounding issue associated with yellow coloration.

Since different types of illumination can affect bee vision (Arnold and Chittka, 2012) conducting experiments across a variety of lighting regimes may be important. However, brightness has generally been found to be an unimportant variable when chromatic differences provide for discrimination. Detecting brightness mandates training (Backhaus, 1991) and appears to matter most for small object identification (Spaethe et al., 2001).

From a behavioral perspective, flower color plays an important role in flower constancy. Flower constant pollinators visit only a subset of the species or morphs extant, despite the presence of alternative, untapped rewards that may be bypassed en route to the preferred resource (Waser, 1986). Flower color also plays an essential role when flower constant bees shift their preference to novel focal phenotypes (Chittka et al., 1997; Chittka et al., 1999). Whether UV floral guide size could contribute to flower constancy remains to be investigated.

In this work, we identified a normal distribution for floral guide size in wild and cultivated populations, though additional data would now be useful to determine whether cultivating plants disrupts the typical floral guide size found in wild plants. It is interesting to note that the mean floral guide size was smaller in the wild population than the cultivated one, and that this wild population had a larger variance in guide size. This comparison raises an important point: traits essential to pollinators can be altered when we cultivate plants, whether we can literally see these traits or not. To address whether, and how much, we alter UV floral guides when we cultivate flowers, more wild and cultivated populations should be surveyed for both UV guide size and for population-level variation in this trait.

Our work also prompts some fundamental questions like ‘Does co-evolution maintain UV vision in bees?’, ‘Does this result in natural selection for the maintenance of UV traits in flowers?’. Retention of UV opsins is ancient and widespread in insects (Briscoe and Chittka, 2001; Kevan et al., 2001), suggesting constraints on evolutionary changes in these genes. Since simple mutations in opsins can shift visual capability, the long-term persistence of UV vision suggests an adaptive value to this trait.

Global declines of commercial and wild bees raise serious pollination-service and economic concerns (Watanabe, 1994; Steffan-Dewenter et al., 2005; Biesmeijer et al., 2006; Brown and Paxton, 2009; Frankie et al., 2009; Gross, 2011; Bommarco et al., 2012; Cameron et al., 2011). This means that advancing our knowledge about the relative attractiveness of conspecific flowers to pollinators is relevant now more than ever. Urban landscapes and sprawl are increasing globally, which decreases the available natural habitat for native pollinations and often replaces it with small, landscaped parcels (Olujimi, 2009). Wild bees are rarely studied in this expanding modern habitat, making experimental work evaluating the pollination behavior of native bees in urban landscapes particularly timely. Recent work has shown that conscientious landscape management can increase bee diversity (Ahrné et al., 2001). Our work now shows that UV absorbent floral guides can play an important role in pollinator recruitment as well.

## MATERIALS AND METHODS

### I. Floral specimens and relevant techniques for measuring UV floral guides

#### Floral specimens

*R. hirta* and *R. fulgida* are Asters. They produce one, large floral head, that is comprised of many central black florets, commonly called true flowers. These tiny true flowers are surrounded by long ligulate florets that function like large petals. They are yellow to humans. Generally, the proximal half of these ‘petals’ is comprised of UV absorptive floral guide. In UV photography, this presents as a bold, dark ring around the center of the entire floral head, where the UV is absorbed. Early work refers to this UV pattern as a ‘nectar guide’ (Daumer, 1958; Eisner et al., 1969; Thompson et al., 1972). The distal portion of these petals is UV reflective and ‘human yellow’. The UV reflectance peaks at ∼360 nm as can be seen on spectrophotometric reflectance graphs (Thompson et al., 1972; Schlangen et al., 2009).

#### Photography and image analysis

UV photography was conducted using a Baader U-filter. This filter transmits UV wavelengths from 325 to 369 nm, with maximal penetrance in the UV-A range at 360 nm (Savazzi, 2011). The filter was used with an AF Micro Nikkor 60 mm lens, several mounts, and a Nikon D70 DSLR digital camera. UV photographs were downloaded to Nikon Capture NX2 software and then transferred to Image J 1.46 for quantitative analyses. Image J is an open access processing and analysis program written in Java that can be downloaded from National Institutes of Health (http://rsbweb.nih.gov/ij). In Image J, a phenotypic trait, like petal surface area can be traced and digitized. The circumscribed area can be calculated and the resultant pixel measurement for the trait can be converted to a meaningful scale (e.g. mm^2^) based upon a reference scale used in the picture (here, a ruler). Multiple traits were measured including petal length, petal surface area, floral-head area, UV floral guide length, and proportion of petal surface area comprised of UV floral guide. All statistical tests were performed in SPSS.

### II. Floral-guide size-distributions for three data sets: greenhouse, urban and wild *R. hirta*

#### Greenhouse flowers

In November 2010, native *R. hirta* seeds were obtained from the Ornamental Plant Germplasm Center of The Ohio State University. In March 2011, 236 seeds were planted under full-spectrum 12L: 12D lighting in the Kaplan Orchid Conservatory of Old Dominion University (ODU, Norfolk, VA, USA, 36.885441N, −76.307466W). After seven weeks, plants were transferred the ODU greenhouse until flowering. In May 2011, flowers were used in a pilot study assessing UV-absorptive floral-guide length (mm) variation. Since plants were grown in the same conditions, variation may represent the genetic component of phenotypic diversity. Floral guides were measured for 18 flowers and two plants and these measurements were compared with an independent samples t-test. The t-test assumption regarding equal variance was met (Levene's test F = 0.00, P = 1.0).

#### Naturalized urban flowers

In July 2010, we assessed the average sized UV absorptive floral guide by measuring the proportion of petal surface area that was comprised of floral guide (mm^2^) relative to the entire petal surface area, for each flower. We also evaluated the distribution of these guide sizes for this naturalized *R. hirta* population. Measurements were made for 30 flowers using Image J on photographs of all flowers. Flowers measured were located in a large, conspecific population on the ODU urban campus.

To evaluate the shape of the distribution for UV floral guide sizes, we used Shapiro-Wilks' test. Consistent with many quantitative traits, the Shapiro-Wilks' test indicated that UV guide sizes were normally distributed in these naturalized flowers (W = 0.984, d.f. = 27, P = 0.938).

Next, we used Pearson's correlation coefficient to determine whether there was a relationship between the floral head width (in mm), and the total size of the floral guide (area in mm^2^), because this allows us to address questions like ‘Is UV guide size correlated with overall flower size?’. Since a significant result could merely represent a positive correlation between the total floral head size and amount of petal surface area that was floral guide, we next considered whether there was a correlation between the total floral head size and the proportion of the total petal surface area that was comprised of floral guide. This allows us to address whether larger floral heads have relatively larger guides (or greater percentage of petal surface area covered by UV guide) than smaller floral heads.

#### Wild flowers

In July 2011, we assessed the floral guide size distribution in the wild flower population for comparison to the naturalized flowers. Thirty flowers growing on a precipitous rocky outcrop in montane Colorado (39.44419N, 105.74084W) were evaluated in the same manner as the naturalized urban flowers. Based upon the Shapiro-Wilks' test, the shape of the distribution of UV floral guide sizes for this population, like the urban one, was also normally distributed (W = 0.958, d.f. = 21, P = 0.467).

### III. Insect visitation response to floral guide manipulations

In the following series of experiments, floral guide size was manipulated in one of two species (*R. hirta* and *R. fulgida*) to determine whether insect visitation rates differed when floral guides were removed, enhanced and diminished. No flowers were reused in any trials.

#### a. Sunscreen masked floral guides on *R. hirta*

In July and August 2010, we conducted a sunscreen study with *R. hirta* from the urban population. Three treatments were used, two new flowers per treatment, for 15 trials. Thus, six floral heads located in the garden were haphazardly identified for use for each trial after visual matching for size and stage. Haphazard selection should randomize error variance. For treatment one, two floral heads were ‘sunscreen’ misted (Ocean Potion Suncare Instant Dry SPF 70 Mist) to mask the floral guide entirely. Treatment two was ‘water misted’ as a control for moisture (the true sunscreen control of sunscreen minus the active ingredient was unavailable). Treatment three was ‘air-sprayed’ as control for potential loss of pollen during water misting.

This study site was intentionally selected to assay the community of insects naturally attracted to cultivars in a large, landscaped urban garden. The garden was situated in front of a five-story building and comprised of a large tract of *R. hirta* (12 m×1.5 m), a smaller tract of *Echinacea purpurrea* (5′×2′) behind the *R. hirta*, and a few *Hosta* cultivars on the garden edges.

All trials were completed on hot summer full-sun days a few days apart from one another. At ∼10:00 am, one hour trials were initiated and the total number of insect visitations was recorded. To be counted, the insect had to alight on a flower head then demonstrate some visual evidence of foraging or pollen collection. First landings were the sole landings counted for the few insects that were observed landing twice so as to avoid repeated measures. Insects were identified for this and subsequent experiments based upon our own expertise and guidebooks (e.g. Arnett and Jacques, 1981; Evans, 2007; Michener et al., 1994; Michener, 2007). Visitation data were analyzed with a Chi-square test and a post-hoc pairwise comparison test that assessed homogeneity of proportions (Cox and Key, 1993). The post-hoc test used the absolute value of the difference between two cell contributions in paired tests. The difference is distributed as Chi square with one degree of freedom so the Δ Chi square between a pair of treatments was compared to 3.84 to assess statistical significance at the P = 0.05 level.

#### b. Augmented floral guides on *R. fulgida*

In August 2010, the first of a series of ‘cut-and-paste’ experiments was conducted in a diverse urban home garden (∼6 m×6 m) in Norfolk, VA. This garden was more complex than the above one, comprised of scores of cultivated *R. fulgida* plants surrounded by a variety of additional flowering perennial species. Treatment *R. fulgida* (Goldsturm) cultivars from a chain store were used in these experiments to evaluate insect response to experimentally diminished and enhanced floral guides.

To create the ‘cut-and-paste’ treatments ∼10 individual floral heads were collected for each trial. All petals were removed from heads, then cut sagittally at the point where the UV floral guide ended (about half way down the petal). UV photographs were taken to determine whether subsequent trimming was necessary to ensure that pieces were entirely floral guide or completely devoid of floral guide. The appropriate petal pieces were glued onto fresh floral heads contingent upon treatment.

Two ‘cut-and-paste’ treatments and one control were freshly constructed for each trial. Treatment one had a ‘diminished’, or nearly no, floral guide and was created by gluing the petal pieces devoid of floral guide atop the proximal portion of an unmanipulated flower head. Treatment two had an ‘enhanced’ or double floral guide, where an additional concentric ring of floral guide was added distally to the natural guide by pasting small floral guide petal pieces on another unmanipulated flower head. For the ‘wild-type cut-and-paste control’ flower, two sets of petal pieces, one with floral guide, and one devoid of it, were glued atop the petals of a final unmanipulated floral head, recreating the common wild-type pattern.

Each floral head (with stalk ∼20 mm) was placed into a clear glass bottle on the ground ∼3 meters away from the conspecific flowerbed. Six trials were conducted from about noon until 2:00 pm for six days and each ended after 10 insect visitations. Treatment locations were rotated in the bottle line-up in subsequent trials (treatment on the left in trial 1 was positioned in the middle for trial 2, and on the right for trial 3, and so forth) such that each trial was comprised of a novel treatment series. Trials were conducted from August 21–27 in Norfolk, VA (36.886246N, −76.289351W). The time, treatment visited, weather, and visitor type were recorded. Visitation data were analyzed with a Chi-square test and a post-hoc pairwise comparison test, as above.

#### c. Slightly enlarged and diminished floral guides on *R. fulgida*

In a less cultivated, more natural setting at an urban sanctuary, a similar ‘cut-and-paste’ experiment was completed. *R. fulgida* were again altered to create two treatments and one ‘wild-type cut-and-paste’ control. Treatment one was ‘enhanced’, here to be slightly enlarged from the natural floral guide to ∼60% of the petal surface area. Treatment two was ‘diminished’, here to be ∼25% of the petal surface. Treatment three was the same ‘wild-type cut-and-paste control’ used previously. Additionally, a ‘natural’ unmanipulated flower head was added as a true control for our ‘cut-and-paste’ procedure. Treatment bottles were positioned near the *Rudbeckia* plot of the ‘meadow flower bed’ at Weyanoke Bird and Wildflower Sanctuary in Norfolk, VA (36.87373N, −76.307068W). This floral bed contained scores of *Rudbeckia*, along with a number of additional annual and perennial meadow flowers, some naturalized. The experiment was conducted from mid-September until mid-October in 2010 from ∼noon until 2:00 pm. Data were recorded and statistics performed as above.

#### d. Oversized floral guides on *R. hirta*

At this point we knew small floral guides were least desirable and we wanted to focus on whether large, uninterrupted guides were preferred. As well, to determine if our results held for more than one species, the native *R. hirta* seed grown in the greenhouse were used for this study. This work was conducted on a grassy knoll, further away (∼25 m) from a smaller, mixed species floral garden at an urban zoo. This site allowed us to determine whether there were differences in the families recruited to this more monoculture-like urban habitat. Treatment one was another enhanced floral guide, here quite oversized, and comprising ∼90% of the petal surface. Treatment two was the ‘wild-type cut-and-paste control’ from above and treatment three was again a natural flower. Nine consecutive trials ran in full sun from ∼10:00 am to 2:00 pm per day in June of 2011. In addition to the Chi-square test and post-hoc tests described above here the cumulative (or total) and mean visitation times were also recorded for all treatments. The variance in mean visitation times was large though homogenous (Levene's statistic = 0.056, d.f._1_ = 2, d.f._2_ = 27, P = 0.946) and these data were square root transformed prior to ANOVA.

This ‘oversized’ treatment experiment was repeated in September of 2011 for seven days. Here, insects were captured with mesh nets and removed after landing to ensure no repeat measures were included in the data set. The variance in mean visitation times was large though homogenous (Levene's statistic = 0.009, d.f._1_ = 2, d.f._2_ = 29, P = 0.992) and these data were log_10_ transformed prior to ANOVA. Before we started this experiment, we conducted a food-color mark and recapture pilot study on Halictidae (*n* = 20) collected during a one-day trial. No marked visitors were identified the subsequent day but food-color marks remained on bees held for 24 hrs.

This ‘oversized’ treatment experiment was repeated again in October of 2011, but here the design was a linear array. Thirty *R. hirta* flower heads (three treatments ×10 flowers per treatment) were positioned ∼60 mm apart from one another in one long array, where treatment order repeated sequentially, and visitation monitored for a longer, single day time period. All of these experiments were conducted at the Virginia Zoo (36.8794675N, −76.274154W) in Norfolk, VA.

## References

[b1] Ahrné K. Bengtsson J. Elmqvist T. (2001). Bumble bees (Bombus spp) along a gradient of increasing urbanization. PLoS ONE 4, e5574 10.1371/journal.pone.0005574 19440367 PMC2679196

[b2] Andersson M. (1982). Female choice selects for extreme tail length in a widowbird. Nature 299, 818–820 10.1038/299818a0

[b3] Arnett R. H. Jr Jacques R. L. Jr (1981). Simon and Schuster's Guide to Insects New York, NY: Simon and Schuster Inc., Fireside Books (Holiday House).

[b4] Arnold S. E. J. Chittka L. (2012). Illumination preference, illumination constancy and colour discrimination by bumblebees in an environment with patchy light. J. Exp. Biol. 215, 2173–2180 10.1242/jeb.065565 22675177

[b5] Backhaus W. (1991). Color opponent coding in the visual system of the honeybee. Vision Res. 31, 1381–1397 10.1016/0042-6989(91)90059-E 1891826

[b6] Backhaus W. Menzel R. Kreißl S. (1987). Multidimensional scaling of color similarity in bees. Biol. Cybern. 56, 293–304 10.1007/BF00319510

[b7] Bennett A. T. Cuthill I. C. (1994). Ultraviolet vision in birds: what is its function? Vision Res. 34, 1471–1478 10.1016/0042-6989(94)90149-X 8023459

[b8] Bennett A. T. D. Cuthill I. C. Partridge J. C. Maier E. J. (1996). Ultraviolet vision and mate choice in zebra finches. Nature 380, 433–435 10.1038/380433a0

[b9] Biesmeijer J. C. Roberts S. P. M. Reemer M. Ohlemüller R. Edwards M. Peeters T. Schaffers A. P. Potts S. G. Kleukers R. Thomas C. D. (2006). Parallel declines in pollinators and insect-pollinated plants in Britain and the Netherlands. Science 313, 351–354 10.1126/science.1127863 16857940

[b10] Bommarco R. Lundin O. Smith H. G. Rundlöf M. (2012). Drastic historic shifts in bumble-bee community composition in Sweden. Proc. R. Soc. B 279, 309–315 10.1098/rspb.2011.0647 PMC3223670 21676979

[b11] Briscoe A. D. Chittka L. (2001). The evolution of color vision in insects. Annu. Rev. Entomol. 46, 471–510 10.1146/annurev.ento.46.1.471 11112177

[b12] Briscoe A. D. Bernard G. D. Szeto A. S. Nagy L. M. White R. H. (2003). Not all butterfly eyes are created equal: rhodopsin absorption spectra, molecular identification, and localization of ultraviolet-, blue-, and green-sensitive rhodopsin-encoding mRNAs in the retina of Vanessa cardui. J. Comp. Neurol. 458, 334–349 10.1002/cne.10582 12619069

[b13] Brown M. J. F. Paxton R. J. (2009). The conservation of bees: a global perspective. Apidologie (Celle) 40, 410–416 10.1051/apido/2009019

[b14] Burkhardt D. (1982). Birds, berries and UV. A note on some consequences of UV vision in birds. Naturwissenschaften 69, 153–157 10.1007/BF00364887 7088195

[b15] Burns J. G. (2005). Impulsive bees forage better: the advantage of quick, sometimes inaccurate foraging decisions. Anim. Behav. 70, e1–e5 10.1016/j.anbehav.2005.06.002

[b16] Burr B. Rosen D. Barthlott W. (1995). Untersuchungen zur Ultraviolettreflexion von Angiospermenblüten. III. Dilleniidae und Asteridae Stuttgart: Franz Steiner.

[b17] Cameron S. A. Lozier J. D. Strange J. P. Koch J. B. Cordes N. Solter L. F. Griswold T. L. (2011). Patterns of widespread decline in North American bumble bees. Proc. Natl. Acad. Sci. USA 108, 662–667 10.1073/pnas.1014743108 21199943 PMC3021065

[b18] Caruso C. M. (2000). Competition for pollination influences selection on floral traits of Ipomopsis aggregata. Evolution 54, 1546–1557. 11108583 10.1111/j.0014-3820.2000.tb00700.x

[b19] Chittka L. Menzel R. (1992). The evolutionary adaptation of flower colours and the insect pollinators' colour vision. J. Comp. Physiol. A 171, 171–181 10.1007/BF00188925

[b20] Chittka L. Beier W. Hertel H. Steinmann E. Menzel R. (1992). Opponent colour coding is a universal strategy to evaluate the photoreceptor inputs in Hymenoptera. J. Comp. Physiol. A 170, 545–563 10.1007/BF00199332 1507155

[b21] Chittka L. Shmida A. Troje N. Menzel R. (1994). Ultraviolet as a component of flower reflections, and the colour perception of Hymenoptera. Vision Res. 34, 1489–1508 10.1016/0042-6989(94)90151-1 8023461

[b22] Chittka L. Gumbert A. Kunze L. (1997). Foraging dynamics of bumble bees: correlates of movements within and between plant species. Behav. Ecol. 8, 239–249 10.1093/beheco/8.3.239

[b23] Chittka L. Thomson J. D. Waser N. M. (1999). Flower constancy, insect psychology, and plant evolution. Naturwissenschaften 86, 361–377 10.1007/s001140050636

[b24] Chittka L. Dyer A. G. Bock F. Dornhaus A. (2003). Psychophysics: bees trade off foraging speed for accuracy. Nature 424, 388 10.1038/424388a 12879057

[b25] Cox M. K. Key C. H. (1993). Post hoc pair-wise comparisons for the chi-square test of homogeneity of proportions. Educ. Psychol. Meas. 53, 951–962 10.1177/0013164493053004008

[b26] Daumer K. (1956). Reizmetrische Untersuchung des Farbensehens der Bienen. Z. Vgl. Physiol. 38, 413–478.

[b27] Daumer K. (1958). Blumenfarben, wie sie die Bienen sehen. Z. Vgl. Physiol. 41, 49–110.

[b28] Dikmen F. (2007). The role and the importance of the family Halictidae (Apiformes: Apoidea) in pollination of natural and agricultural vegetation. Mellifera 7, 16–19.

[b29] Dyer A. G. (1996). Reflection of near-ultraviolet radiation from flowers of Australian native plants. Australian Journal of Botany 44, 473–488 10.1071/BT9960473

[b30] Dyer A. G. Spaethe J. Prack S. (2008). Comparative psychophysics of bumblebee and honeybee colour discrimination and object detection. J. Comp. Physiol. A 194, 617–627 10.1007/s00359-008-0335-1 18437390

[b31] Dyer A. G. Paulk A. C. Reser D. H. (2011). Colour processing in complex environments: insights from the visual system of bees. Proc. R. Soc. B 278, 952–959 10.1098/rspb.2010.2412 PMC3049058 21147796

[b32] Eisner T. (2002). An insect's view of a flower. American Entomologist 48, 142–143.

[b33] Eisner T. Silberglied R. E. Aneshansley D. Carrel J. E. Howland H. C. (1969). Ultraviolet video-viewing: the television camera as an insect eve. Science 166, 1172–1174 10.1126/science.166.3909.1172 17775577

[b34] Evans A. V. (2007). National Wildlife Federation Field Guide to Insects and Spiders of North America New York, NY: Sterling Publishing.

[b35] Frankie G. W. Rizzardi M. Vinson S. B. Griswold T. L. (2009). Decline in bee diversity and abundance from 1974–2004 on a flowering leguminous tree, Andira inermis in Costa Rica at the interface of disturbed dry forest and the urban environment. J. Kans. Entomol. Soc. 82, 1–20 10.2317/JKES708.23.1

[b36] Giurfa M. Vorobyev M. (1998). The angular range of achromatic target detection by honey bees. J. Comp. Physiol. A 183, 101–110 10.1007/s003590050238

[b37] Giurfa M. Núñez J. Chittka L. Menzel R. (1995). Colour preferences of flower-naive honeybees. J. Comp. Physiol. A 177, 247–259 10.1007/BF00192415

[b38] Giurfa M. Vorobyev M. Kevan P. Menzel R. (1996). Detection of coloured stimuli by honeybees: minimum visual angles and receptor specific contrasts. J. Comp. Physiol. A 178, 699–709 10.1007/BF00227381

[b39] Giurfa M. Vorobyev M. Brandt R. Posner B. Menzel R. (1997). Discrimination of coloured stimuli by honeybees: alternative use of achromatic and chromatic signals. J. Comp. Physiol. A 180, 235–243 10.1007/s003590050044

[b40] Gross M. (2011). New fears over bee declines. Curr. Biol. 21, R137–R139 10.1016/j.cub.2011.02.001 21438183

[b41] Höglund G. Hamdorf K. Rosner G. (1973). Trichromatic visual system in an insect and its sensitivity control by blue light. J. Comp. Physiol. A 86, 265–279 10.1007/BF00696344

[b42] Johnson S. D. Andersson S. (2002). A simple field method for manipulating ultraviolet reflectance of flowers. Can. J. Bot. 80, 1325–1328 10.1139/b02-116

[b43] Jones C. E. Buchmann S. L. (1974). Ultraviolet floral patterns as functional orientation cues in Hymenopterous pollination systems. Anim. Behav. 22, 481–485 10.1016/S0003-3472(74)80047-3

[b44] Kevan P. G. (1978). Floral coloration, its colorimetric analysis and significance in anthecology. The Pollination of Flowers by Insects (Linnaean Society Symposium Series) Richards A J, ed51–78 London: Linnaean Society.

[b46] Kevan P. G. Chittka L. Dyer A. G. (2001). Limits to the salience of ultraviolet: lessons from colour vision in bees and birds. J. Exp. Biol. 204, 2571–2580. 11511673 10.1242/jeb.204.14.2571

[b47] Kien J. Menzel R. (1977a). Chromatic properties of interneurons in the optic lobes of the bee. I. Broad band neurons. J. Comp. Physiol. A 113, 17–34 10.1007/BF00610451

[b48] Kien J. Menzel R. (1977b). Chromatic properties of interneurons in the optic lobe of the bee. II. Narrow band and colour opponent neurons. J. Comp. Physiol. A 113, 35–53 10.1007/BF00610452

[b49] Lehrer M. Srinivasan M. V. Zhang S. W. (1990). Visual edge detection in the honeybee and its chromatic properties. Proc. R. Soc. B. 238, 321–330 10.1098/rspb.1990.0002

[b50] McCrea K. D. Levy M. (1983). Photographic visualization of floral colors as perceived by honeybee pollinators. Am. J. Bot. 70, 369–375 10.2307/2443245

[b51] Menzel R. Shmida A. (1993). The ecology of flower colours and the natural colour vision of insect pollinators: The Israeli flora as a study case. Biol. Rev. Camb. Philos. Soc. 68, 81–120 10.1111/j.1469-185X.1993.tb00732.x

[b52] Menzel R. Backhaus W. (1991). Colour vision in insects. Vision and Visual Dysfunction: The Perception of Colour, Vol. 6 Gouras P, ed262–293 Houndsmills: MacMillan Press.

[b53] Menzel R. Blakers M. (1976). Colour receptors in the bee eye – morphology and spectral sensitivity. J. Comp. Physiol. A 108, 11–13.

[b54] Michener C. D. (2007). The Bees of the World, 2nd edition Baltimore, MD: The Johns Hopkins University Press.

[b55] Michener C. D. McGinley R. J. Danforth B. N. (1994). The Bee Genera of North and Central America (Hymenoptera: Apoidea) Washington, DC: Smithsonian Institution Press.

[b56] Olujimi J. (2009). Evolving a planning strategy for managing urban sprawl in Nigeria. J. Hum. Ecol. 25, 201–208.

[b57] Peitsch D. Backhaus W. Menzel R. (1989). Color vision systems in Hymenopterans: a comparative study. Neural Mechanisms of Behavior Erber J Menzel R Pflüger H J Todt D, ed163 Stuttgart; New York, NY: Thieme.

[b58] Peitsch D. Fietz A. Hertel H. de Souza J. Ventura D. F. Menzel R. (1992). The spectral input systems of hymenopteran insects and their receptor-based colour vision. J. Comp. Physiol. A 170, 23–40 10.1007/BF00190398 1573568

[b59] Reser D. H. Wijesekara Witharanage R. Rosa M. G. P. Dyer A. G. (2012). Honeybees (Apis mellifera) learn color discriminations via differential conditioning independent of long wavelength (green) photoreceptor modulation. PLoS ONE 7, e48577 10.1371/journal.pone.0048577 23155394 PMC3498261

[b60] Rohde K. Papiorek S. Lunau K. (2013). Bumblebees (Bombus terrestris) and honeybees (Apis mellifera) prefer similar colours of higher spectral purity over trained colours. J. Comp. Physiol. A 199, 197–210 10.1007/s00359-012-0783-5 23224278

[b61] Savazzi E. (2011). Digital Photography for Science: Close-up Photography, Macrophotography and Photomacrography Raleigh, NC: Lulu.com.

[b62] Schlangen K. Miosic S. Castro A. Freudmann K. Luczkiewicz M. Vitzthum F. Schwab W. Gamsjäger S. Musso M. Halbwirth H. (2009). Formation of UV-honey guides in Rudbeckia hirta. Phytochemistry 70, 889–898 10.1016/j.phytochem.2009.04.017 19477473

[b63] Silberglied R. E. (1979). Communication in the ultraviolet. Annu. Rev. Ecol. Syst. 10, 373–398 10.1146/annurev.es.10.110179.002105

[b64] Skorupski P. Chittka L. (2010). Photoreceptor spectral sensitivity in the bumblebee, Bombus impatiens (Hymenoptera: Apidae). PLoS ONE 5, e12049 10.1371/journal.pone.0012049 20711523 PMC2919406

[b65] Spaethe J. Tautz J. Chittka L. (2001). Visual constraints in foraging bumblebees: flower size and color affect search time and flight behavior. Proc. Natl. Acad. Sci. USA 98, 3898–3903 10.1073/pnas.071053098 11259668 PMC31150

[b66] Srinivasan M. V. Lehrer M. (1984). Temporal acuity of honeybee vision: behavioural studies using moving stimuli. J. Comp. Physiol. A 155, 297–312 10.1007/BF00610583

[b67] Stanton M. L. Snow A. A. Handel S. N. Bereczky J. (1989). The impact of a flower-color polymorphism on mating patterns in experimental populations of wild radish (Raphanus raphanistrum L.). Evolution 43, 335–346 10.2307/2409211 28568562

[b68] Steffan-Dewenter I. Potts S. G. Packer L. (2005). Pollinator diversity and crop pollination services are at risk. Trends Ecol. Evol. 20, 651–652, author reply 652-653 10.1016/j.tree.2005.09.004 16701452

[b69] Thompson W. R. Meinwald J. Aneshansley D. Eisner T. (1972). Flavonols: pigments responsible for ultraviolet absorption in nectar guide of flower. Science 177, 528–530 10.1126/science.177.4048.528 5050486

[b70] von Frisch K. (1914). Der Farbensinn und Formensinn der Biene. Zool. Jb. Abt. Allg. Zool. Physiol. 35, 1–182.

[b71] von Frisch K. (1967). The Dance Language and Orientation of Bees Cambridge, MA: Belknap Press of Harvard University Press.

[b72] Waser N. M. (1986). Flower constancy: definition, cause, and measurement. Am. Nat. 127, 593–603 10.1086/284507

[b73] Waser N. M. Price M. V. (1981). Pollinator choice and stabilizing selection for flower color in Delphinium nelsonii. Evolution 35, 376–390 10.2307/2407846 28563376

[b74] Waser N. M. Price M. V. (1985). The effect of nectar guides on pollinator preference: experimental studies with a montane herb. Oecologia 67, 121–126 10.1007/BF00378462 28309856

[b75] Watanabe M. E. (1994). Pollination worries rise as honey bees decline. Science 265, 1170 10.1126/science.265.5176.1170 17787573

[b76] Winter Y. López J. Von Helversen O. (2003). Ultraviolet vision in a bat. Nature 425, 612–614 10.1038/nature01971 14534585

